# Training support, public service motivation, and career retention intentions: the mediating role of job wellbeing among college graduate volunteers in China's rural revitalization

**DOI:** 10.3389/fpsyg.2026.1835086

**Published:** 2026-05-29

**Authors:** Yan Peng, Xue Yang

**Affiliations:** Business School, Huanggang Normal University, Huanggang, China

**Keywords:** career retention intentions, job wellbeing, public service motivation, rural revitalization, training support, volunteerism

## Abstract

**Background:**

The effective implementation of China's rural revitalization strategy relies on a stable young volunteer corps, whose career retention directly impacts volunteer team stability and service efficacy. Public service motivation, a core dimension of volunteers' emotional resilience, and job wellbeing are key to career retention, while training support is a critical exogenous factor, yet their integrated influence mechanism on rural revitalization volunteers' career retention intentions remains underexplored.

**Methods:**

Integrating Social Exchange Theory, Theory of Planned Behavior and Public Service Motivation Theory, this study built a framework of exogenous training support-endogenous public service motivation-job wellbeing-sustained career retention intention. Based on survey data of 369 volunteers from Hubei's University Student Volunteering Program (Rural Revitalization Project)-a nationally standardized program initiated in 2022 in this typical central China province, ordered logistic regression and Bootstrap mediation effect tests were used to examine the core variable influence mechanisms.

**Results:**

Training support and public service motivation both exerted significant positive effects on career retention intentions, with training support having a stronger total effect. Job wellbeing partially mediated the training support-career retention link (mediation contribution: 57.8%), but its mediating role in the public service motivation-career retention relationship was non-significant, with public service motivation driving career retention directly.

**Conclusions:**

This study identified a “dual-path” mechanism for rural revitalization volunteers' career retention, clarifying the differential roles of training support and public service motivation (emotional resilience). The findings provide theoretical basis and practical pathways for optimizing volunteer training and incentive systems, shaping young volunteers' emotional resilience, and consolidating talent support for rural revitalization, while linking training support to career retention (employability) via key mediators of emotional resilience and job wellbeing.

## 1 Introduction

Rural revitalization constitutes a core strategy in the new era for addressing challenges related to agriculture, rural areas, and farmers, as well as for promoting integrated urban-rural development. As crucial agents delivering intellectual capital and on-the-ground services, young volunteers' willingness to sustain their participation directly determines the long-term viability of rural revitalization volunteer services, and is a key indicator of their employability and career development in the grassroots public service field. Since the initiation of the University Student Volunteering Program (Rural Revitalization Project) in Hubei Province in 2022, a comprehensive service system has gradually been established, spanning domains such as rural education, rural development, rural healthcare, rural social governance, and grassroots youth work. Through methods of “open recruitment, voluntary registration, organizational selection, and centralized assignment,” this program has injected significant youthful energy into rural revitalization, and provided a critical career transition platform for college graduates to build emotional resilience and accumulate professional competencies. The essence of volunteerism is a mutually reinforcing dynamic (Tan, 2020), wherein volunteers contribute their professional skills and dedication to rural development, while rural areas, in turn, provide a platform for volunteers' capacity building and value realization, thereby fostering a virtuous cycle of interaction.

Within this interactive process, which factors influence volunteers' career retention intentions for sustained service? Furthermore, what are the differing pathways through which these factors operate? Addressing these questions is crucial for optimizing the management of such specialized programs, achieving a virtuous cycle of “dedication–empowerment–re-dedication,” and constitutes a significant agenda for both rural revitalization talent management and volunteer service research. Accordingly, this paper focuses on the following core research questions. First, at the level of exogenous support, what are the underlying mechanisms through which training support—a key pillar of this specialized program—influences volunteers' career retention intentions for sustained service? Does it exert a direct effect, or is its influence mediated through intermediate psychological perceptions? Second, at the level of endogenous motivation, does public service motivation, as a core psychological driver for volunteers participating in rural revitalization, have a direct impact on their career retention intentions? Is this impact contingent upon mediating variables such as job wellbeing? Third, are there differentiated mechanisms through which training support and public service motivation influence career retention intentions? How can targeted policies be designed to simultaneously amplify the positive effects of both categories of factors, thereby enhancing the stability of volunteer sustained service?

Existing research has accumulated considerable insights into volunteers' career retention intentions; however, several research gaps warrant further investigation. Regarding theoretical perspectives, most studies predominantly rely on unitary theoretical frameworks, lacking a systematic integration of “exogenous institutional support and endogenous psychological motivation.” This limitation hinders a comprehensive understanding of the differentiated influence mechanisms of various antecedent variables. In terms of mediating mechanism research, investigations into the mediating effects of key psychological variables such as job wellbeing remain relatively superficial, failing to adequately address their heterogeneous roles across different influence pathways. Moreover, existing studies largely focus on generalized volunteering contexts, with insufficient adaptation to the specificities of targeted rural revitalization programs. Concerning empirical contexts, there is a paucity of targeted research on regional specialized volunteer services such as Hubei Province's Rural Revitalization Project, making it difficult to capture the particularities and complexities of volunteers' career retention within local policy contexts.

To address these gaps, this study focuses on university student rural revitalization volunteers in Hubei Province, integrating three classical theories to construct a comprehensive analytical framework. Training support and public service motivation are selected as core explanatory variables, job wellbeing as a mediating variable, and policy perception, economic support, and family characteristics as control variables. Employing ordered logistic regression and Bootstrap mediation analysis, this study empirically examines the influence pathways of core variables on career retention intentions, with particular emphasis on revealing the differentiated mediating role of job wellbeing. The marginal contributions of this study are threefold: first, by incorporating the policy design of Hubei Province's Rural Revitalization Project implementation plan, it constructs a dual-core analytical framework of “exogenous training support—endogenous public service motivation,” thereby addressing the limitations of single-factor analyses in existing research. Second, it empirically identifies the heterogeneous mediating effects of job wellbeing within the pathways of the two core variables, deepening the understanding of career retention mechanisms in targeted rural revitalization volunteer services. Third, grounded in the actual implementation context of Hubei Province, it proposes targeted incentive strategies centered on the core variables, offering practical references for local governments to optimize volunteer service management policies, and for enhancing the job wellbeing and grassroots employability of college graduate volunteers.

Beyond China, rural volunteer retention has emerged as a critical challenge in many developing and developed economies. In sub-Saharan Africa, for example, community health volunteers (CHVs) play an indispensable role in delivering primary healthcare to underserved rural populations, yet high turnover rates remain a persistent challenge, with retention strategies often focusing on income-generating activities to reduce attrition (Riang'a and Nyanja, 2024; Lusambili and Nyanja, 2021). Similarly, in rural Kenya, volunteer farmer trainers (VFTs) have been shown to sustain high retention rates-up to 80%-when programs provide not only financial incentives but also entrepreneurial opportunities and a sense of ownership (World Agroforestry Centre, 2014). Across OECD countries, volunteerism is recognized as a key driver of local development, contributing to social cohesion, neighborhood revitalisation, and community resilience to societal pressures such as natural disasters (OECD, 2024). These international experiences highlight a common theme: the interplay between exogenous support (training, incentives, capacity building) and endogenous motivation (value identification, sense of purpose) is critical for volunteer retention. However, existing international research has largely focused on either economic incentives or organizational management, with less attention paid to the integrated mechanism linking training support, public service motivation, and job wellbeing-particularly in the context of government-initiated, nationally standardized volunteer programs. This study addresses this gap by examining the distinct pathways through which training support (exogenous) and public service motivation (endogenous) influence career retention intentions among young volunteers in China's Western Program (Rural Revitalization Project).

## 2 Literature review

### 2.1 Factors influencing volunteer career retention

Existing research has developed a binary analytical framework encompassing “external environment—internal psychology” to examine factors influencing volunteers' career retention intentions. Regarding external environmental factors, organizational management and policy support constitute critical variables affecting volunteer career retention. Conn et al. (2004) found that support related to professional skills and interests, along with cultural and institutional support, are key factors in sustaining student volunteer behavior. Cruce and Moore (2007) similarly demonstrated through surveys that volunteer organization type, as an important component of the external environment, significantly influences students' sustained volunteer participation. (Locke et al., 2003; Studer and von Schnurbein, 2013) argued that clear, developmental, supportive, and appreciative organizational management approaches may encourage volunteers to remain and continue providing services. Conversely, poor volunteer management—including inadequate supervision, limited communication, insufficient training, inappropriate task matching, and failure to respond to volunteers' perceived low value or negative emotions—may lead volunteers to leave volunteer organizations. De Waele and Hustinx (2018) suggested that in modern societies, governments actively promote volunteerism development through legislation, competitive initiatives, supportive actions, and incentives based on various rationales. Zhang and Wu (2015) found that factors including volunteer service organizations' basic capabilities, their service attitudes toward volunteers and service recipients, and service effectiveness significantly influence volunteer service discontinuation, with organizational factors being key determinants. Zhang and Zhu (2018) discovered that external factors such as university and government support, organizational management satisfaction, and job placement satisfaction significantly influence deep participation levels. He and Qi (2020) found that standardized and professionalized volunteer service management significantly positively influences volunteers' career retention intentions. Liu and Li (2023) argued that volunteer service supply sustainability is closely related to three factors: individual volunteer capabilities, organizational incentives for individuals, and resources individuals obtain from organizations. Huang and Lin (2024) revealed that the “project mobilization” model effectively integrates administrative and social forces, promoting youth engagement at the grassroots level.

Regarding internal psychological factors, public service motivation and value identification constitute core drivers of volunteer career retention, with domestic and international research examining these from diverse perspectives. International scholarship on volunteer motivation classification is relatively mature. Ziemek (2006) categorized volunteer motivations into altruistic, egoistic, and investment motivations, providing a foundational framework for understanding motivation's influence on career retention. (Omoto and Snyder (1995), from a psychological perspective, emphasized that volunteers' individual motivations, needs, and personal volunteer experiences are key to behavioral continuity in volunteerism. Matsuba et al. (2007) constructed a multi-factor model categorizing influencing factors into enduring and moderating types, confirming that volunteer behavioral continuity results from multiple interacting factors, with enduring factors exerting influence through moderating factors. (Wondimu and Admas (2024) suggested that desire for learning experiences (enhancing resumes) and applied skills (professional capacity development) are primary motivations for student volunteer participation. In domestic research, Luo and Wang (2012) found that volunteer activity participants possess both “altruistic” and “egoistic” motivations, with career retention influenced by their social networks. Additionally, volunteers' self-identification with their roles and incentive systems within group contexts may promote their sense of mission and continuous service provision. Ng et al. (2019) found that volunteers with altruistic motivations demonstrate stronger initiative and autonomy in volunteering. Wang and Wei (2020) categorized volunteers into enthusiastic, growth-oriented, dormant, and passive types based on participation intentions and behavioral sustainability. Cui (2022) empirical research confirmed that public sense of social responsibility, national-level values, and individual-level values significantly positively influence volunteer participation, with actual volunteer service efficacy evaluation being highly correlated with participation sustainability.

### 2.2 The distinctive nature of rural revitalization volunteer services

University student volunteerism in rural revitalization constitutes a form of prosocial behavior wherein student volunteers, leveraging their acquired knowledge and skills, provide services to advance rural development (Hu, 2023). This engagement offers significant potential for fostering rural economic and social progress (Wu, 2024). Rural revitalization volunteer services exhibit significant differences from general volunteerism due to characteristics including complex service contexts, clear task orientations, and strong policy relevance, resulting in unique management mechanisms and career retention logic. The logic of “altruistic reciprocity” is particularly prominent in rural revitalization volunteer services: while dedicating themselves to rural areas, volunteers also emphasize personal capacity enhancement and value realization (Hu, 2025). Zhou and Zou (2021) found that gender, grade level, institution type, level of understanding, and volunteer organizations significantly influence university students' participation in rural volunteer services. Zhang and Yuan (2022) identified that university student rural revitalization volunteer service project operations are primarily realized through four mechanisms: recruitment and mobilization, training and cultivation, guarantee and incentives, and evaluation and transformation. Lin and Zhou (2025), focusing on university student participation in rural revitalization services, proposed establishing a multi-stakeholder collaborative guarantee system involving government, universities, rural areas, and enterprises, addressing the practical challenge of insufficient volunteer service sustainability through precise supply-demand matching, long-term cooperation, and bidirectional empowerment. Yang and Lin (2026) found that volunteers serving at the grassroots level face multiple challenges including life adaptation and cultural integration, imposing higher demands on volunteer service organizational management and support systems.

### 2.3 Research gaps and study opportunities

In summary, existing research has established a solid foundation for understanding volunteer career retention behavior. However, considering the contextual characteristics of targeted rural revitalization volunteer services and this study's core research questions, three areas warrant further exploration. First, theoretical integration remains insufficient. Most studies focus on single influencing factors or unitary theoretical perspectives, lacking systematic integration of the complete “external support—internal motivation—psychological mediation” chain, which hinders comprehensive understanding of the complex mechanisms underlying rural revitalization volunteer career retention. Second, contextual adaptation is inadequate. Existing research insufficiently addresses the policy context and service characteristics of targeted rural revitalization programs, with a particular paucity of empirical studies on regional targeted programs such as Hubei Province's initiative, making it difficult to capture the particularities of volunteer career retention within local policy designs. Third, research on mediating mechanisms lacks depth. Insufficient attention has been paid to the heterogeneous roles of key psychological variables such as job wellbeing across different antecedent pathways, failing to clarify their differentiated functions in how external support and internal motivation influence career retention intentions.

Addressing these gaps, this study takes Hubei Province's University Student Volunteering Program (Rural Revitalization Project) as its specific research context, with the practical challenge of volunteer retention during program implementation as its empirical starting point. Integrating Social Exchange Theory, the Theory of Planned Behavior, and Public Service Motivation Theory, it constructs an integrated analytical framework focusing on the differentiated influence mechanisms of training support and public service motivation, aiming to address existing research limitations and provide theoretical and empirical support for local governments' optimization of volunteer service policies.

## 3 Theoretical foundation and research hypotheses

### 3.1 Theoretical foundation

This study focuses on the practical context of Hubei Province's University Student Volunteering Program (Rural Revitalization Project) and integrates Social Exchange Theory (SET), the Theory of Planned Behavior (TPB), and Public Service Motivation Theory (PSM) to construct an integrated analytical framework of “exogenous training support—endogenous psychological motivation—mediating transmission—career retention intention.” This framework aims to reveal the differentiated influence logic of various antecedent variables on volunteers' career retention intentions.

#### 3.1.1 Social exchange theory (SET)

Social Exchange Theory posits that individual behavior fundamentally constitutes a process of resource exchange, wherein individuals evaluate their “investments” against “returns” during interactions. When perceived returns equal or exceed investments, individuals tend to maintain the exchange relationship; conversely, they may terminate it. In the context of rural revitalization volunteer services, volunteers' “investments” manifest as contributions of time, energy, and professional skills, while “returns” encompass both exogenous resource gains—such as training support and policy guarantees—and endogenous psychological benefits—including job wellbeing and self-value realization. This logic of “altruistic reciprocity” is particularly salient in rural revitalization volunteerism (Hu, 2025). According to the 2024–2025 Implementation Plan for the Hubei Province University Student Volunteering Program (Rural Revitalization Project), training support constitutes one of the program's core guarantee measures, encompassing both preservice centralized training and in-service specialized training, designed to enhance volunteers' professional competencies and adaptive capabilities. When training support effectively aligns with rural service demands, it reduces service difficulty and enhances work efficacy, enabling volunteers to perceive a positive exchange between “service investment” and “capacity returns,” thereby strengthening their motivation for career retention. Conversely, insufficiently targeted training may render volunteers incapable of fulfilling service tasks, fostering perceptions of imbalance characterized by “high investment—low returns” and consequently suppressing career retention intentions.

#### 3.1.2 Theory of planned behavior (TPB)

The Theory of Planned Behavior posits that individual behavioral intention is driven by three core factors: (a) attitude toward the behavior (subjective evaluation of the behavior, e.g., job wellbeing); (b) subjective norms (internalized social values and expectations, e.g., public service motivation); and (c) perceived behavioral control (judgments regarding behavioral feasibility and controllability, e.g., institutional support). This theory provides a framework for integrating the logical chain of “exogenous support—endogenous motivation—behavioral intention.” Within Hubei Province's Rural Revitalization Project, training support enhances volunteers' “perceived behavioral control” over rural service tasks by providing professional skills and practical guidance, thereby increasing their confidence in accomplishing service objectives. Public service motivation, as an internalization of “subjective norms,” manifests as the alignment between volunteers' personal willingness and societal expectations of “responding to national calls and serving rural revitalization.” Job wellbeing, as the core “attitude toward the behavior,” translates external support and internal motivation into stable behavioral intentions. These three factors collectively influence career retention decisions. Furthermore, TPB's emphasis on “contextual adaptability” aligns with the contextual characteristics of rural revitalization volunteer services, effectively explaining how situational factors—such as policy environments and service tasks—influence intention formation.

#### 3.1.3 Public service motivation theory (PSM)

Public Service Motivation Theory emphasizes that individuals' core motivation for participating in public affairs originates from intrinsic value identification and public interest orientation, rather than solely from external rewards. Public service motivation reflects the emotional resilience of college graduate volunteers in adapting to the stressful environment of rural grassroots work, which is a trainable psychological capability shaped by formal training and on-the-job practice, and is critical for maintaining subjective job wellbeing and career development intentions. Within Hubei Province's Rural Revitalization Project, motivations such as “responding to national calls, cherishing one's hometown, and serving rural development” fundamentally represent concrete manifestations of public service motivation, reflecting volunteers' intrinsic beliefs regarding “serving the public interest and realizing personal value.” These beliefs possess no table “transcendent” qualities, remaining relatively impervious to short-term fluctuations in external factors such as working conditions or material rewards. Luo and Wang (2012) confirmed that altruism-oriented public service motivation generates stable motivation for career retention. Moreover, the formation and reinforcement of public service motivation are closely intertwined with policy guidance. In the context of Hubei Province's Rural Revitalization Project, the explicit selection criteria—emphasizing “firm ideals and convictions” and a “commitment to grassroots engagement”—serve as a critical policy orientation. This approach further activates volunteers' public service motivation, thereby fostering a direct causal logic from “value identification to behavioral intention.”

#### 3.1.4 Integrated analytical framework

Based on the three aforementioned theories and considering the policy design and practical characteristics of Hubei Province's University Student Rural Revitalization Volunteering Program, this study constructs the following analytical framework: (a) Exogenous pathway: training support, as the program's core institutional support, directly influences career retention intentions by enhancing volunteers' perceived behavioral control. Simultaneously, training support indirectly drives career retention intentions by improving service experiences and enhancing work competencies, thereby increasing job wellbeing (behavioral attitude). (b) Endogenous pathway: public service motivation, as internalized subjective norms, directly drives career retention intentions; its transmission pathway through job wellbeing is nonsignificant due to the directness of value identification. (c) Control pathway: variables including policy perception, economic support, and family characteristics are incorporated to exclude confounding factors' interference with core conclusions. Policy perception is measured based on volunteers' awareness of core policies, while economic support and family support follow the core control variable systems established in existing research.

### 3.2 Research hypotheses

#### 3.2.1 Main effect hypothesis: training support and career retention intentions

From the Social Exchange Theory perspective, training support constitutes a “capacity-enhancing resource” acquired by volunteers. Hubei Province's Rural Revitalization Project explicitly stipulates a “general competence + specialized skills” training system, effectively reducing the professional threshold and work difficulty associated with rural services. This enables volunteers to perceive a positive exchange between “service investment” and “capacity returns,” thereby shaping their emotional resilience and strengthening motivation for career retention. From the Theory of Planned Behavior perspective, training support enhances volunteers' “perceived behavioral control,” increasing their confidence in accomplishing rural revitalization service tasks and directly strengthening behavioral intentions for career retention. Based on this rationale, Hypothesis one is proposed.

**H1:** training support has a significant positive effect on rural revitalization volunteers' career retention intentions.

#### 3.2.2 Main effect hypothesis: public service motivation and career retention intentions

From the Public Service Motivation Theory perspective, “responding to national calls, cherishing one's hometown, and embracing volunteerism” represent volunteers' intrinsic value identification with the rural revitalization strategy. This value-based conviction directly motivates career retention behavior, independent of external rewards. From the Theory of Planned Behavior perspective, public service motivation represents deep internalization of “subjective norms,” aligning both with national rural revitalization strategic orientations and the volunteer spirit of “dedication, fraternity, mutual assistance, and progress.” This dual identification directly translates into behavioral intentions for career retention. Examining Hubei Province's program implementation, the selection criteria emphasizing “firm ideals and beliefs, commitment to grassroots work” further reinforce the positive association between public service motivation and career retention intentions, rendering volunteers with high motivation more likely to develop stable retention intentions. Based on this rationale, Hypothesis two is proposed.

**H2:** public service motivation has a significant positive effect on rural revitalization volunteers' career retention intentions.

#### 3.2.3 Mediation hypothesis: the mediating role of job wellbeing

From the Social Exchange Theory perspective, training support (exogenous resources) cannot directly influence behavioral intentions without being transformed into “psychological returns”—namely, job wellbeing. High-quality training enhances work competencies and reduces frustration, thereby strengthening positive evaluations of service experiences and forming a “resources—psychology—behavior” transmission chain. Specifically, Hubei Province's targeted training helps volunteers rapidly adapt to rural service contexts, enhances professional skill application capabilities, reduces work-related frustration, and consequently fosters positive evaluations of volunteer services (job wellbeing).

From the Theory of Planned Behavior perspective, job wellbeing constitutes the core manifestation of “behavioral attitude.” Training support, by enhancing job wellbeing (positive attitude), enables volunteers to transition from “passive participation” to “active continuation,” ultimately forming a “training support → job wellbeing → career retention intentions” transmission chain. Simultaneously, the direct effect of training support remains present, thus constituting partial mediation. Based on this rationale, Hypothesis three is proposed.

**H3:** job wellbeing partially mediates the relationship between training support and rural revitalization volunteers' career retention intentions.

From the Public Service Motivation Theory perspective, public service motivation originates from intrinsic value identification, and its driving effect on career retention intentions operates directly, independent of transmission through psychological experiences such as job wellbeing.

From the Theory of Planned Behavior perspective, volunteers with strong public service motivation focus more on rural revitalization's actual outcomes and personal value realization. Satisfaction of these higher-order needs does not depend on transmission through job wellbeing; consequently, job wellbeing's mediating role is nonsignificant, with public service motivation influencing career retention intentions primarily through direct effects. Based on this analysis, Hypothesis four is proposed.

**H4:** job wellbeing does not significantly mediate the relationship between public service motivation and rural revitalization volunteers' career retention intentions; public service motivation influences career retention intentions primarily through direct effects.

## 4 Data and research design

### 4.1 data sources and sample characteristics

The data for this study were derived from a 2024 survey conducted among participants in the University Student Volunteering Program (Rural Revitalization Project) in Hubei Province. A stratified sampling strategy was employed, encompassing volunteers across four prefecture-level cities and regions in Hubei—Xianning, Enshi, Shiyan, and Xiangyang—who were engaged in various specialized areas including rural development, rural social governance, and grassroots youth work. A total of 400 questionnaires were distributed, with 369 valid responses obtained, yielding a valid response rate of 92.25%.

Regarding the basic characteristics of the sample: in terms of political affiliation, 27.30% were members of the Communist Party of China (including probationary members), 68.65% were members of the Communist Youth League, and 4.05% were non-affiliated masses. Concerning service duration, volunteers who had completed 1 year of service accounted for 58.26%, while those with less than 1 year of service constituted 41.74%. With respect to service specialization, volunteers engaged in rural development accounted for 44.86%, rural social governance for 40.00%, grassroots youth work for 15.68%, rural education for 10.81%, and rural healthcare for 2.16%. The sample structure closely approximates the overall characteristics of rural revitalization volunteers in Hubei Province, demonstrating strong representativeness.

### 4.2 Variable definition and measurement

#### 4.2.1 Dependent variable: career retention intentions (retention)

This variable was measured using an ordered categorical scale: 1 = reluctant to continue, hoping to shorten or terminate the service period early; 2 = still considering, needing more time to decide; 3 = relatively satisfied, but the originally planned service period is sufficient; 4 = very willing, hoping to extend the service period.

#### 4.2.2 Core explanatory variables

Training Support (sup_train): measured by volunteers' satisfaction with pre-service and in-service training. Responses were coded as 1 = average, 2 = basically satisfied, 3 = very satisfied.

Public Service Motivation (mot_public): operationalized as a summative index ranging from 0 to 3, based on the sum of three motivational items: “responding to national calls,” “cherishing one's hometown,” and “passion for volunteer work.” This continuous measure captures the intensity of volunteers' value-based commitment to public service.

#### 4.2.3 Mediating variable: job wellbeing

Assessed by volunteers' overall evaluation of their volunteer service experience. This single-item measure captures the volunteer's global, evaluative judgment of their wellbeing in the service role, consistent with the conceptualization of subjective wellbeing in organizational research (Diener, 1984). Single-item global measures of wellbeing have been shown to correlate strongly with multi-item scales and predict behavioral outcomes such as retention (Wanous et al., 1997). Responses were coded as 1 = average, 2 = basically satisfied, 3 = very satisfied.

#### 4.2.4 Control variables

Policy Perception (sup_policy): as the questionnaire did not include items specifically measuring satisfaction with policies, this study measured the level of policy support based on volunteers' awareness and understanding of core rural revitalization program policies. Four key policies were selected as measurement items: “service period and working years recognition policy,” “postgraduate admission preferential policy,” “tuition compensation and student loan reimbursement policy,” and “professional title evaluation preferential policy.” A counting assignment method was employed: understanding one policy was assigned a value of one, two policies a value of two, three policies a value of three, and four policies a value of four. Higher values indicate greater awareness and understanding of these policies, reflecting more fully perceived policy support. In research on policy perception in volunteerism and public policy, policy awareness is considered a fundamental prerequisite for perceived policy support.

Economic Support (sup_econ), Security Support (sup_security), Political Affiliation (politics), Family Economic Status (family_econ), Family Support (family_sup), and Service Specialty (service) are presented in Table 1.

**Table 1:** Main variable names and definitions.

Variable type	Variable name	Variable symbol	Definition and assignment	Variable attribute
Dependent variable	career retention intentions	retention	1 = hopes to shorten the service period; 2 = undecided; 3 = original service period is sufficient; 4 = hopes to extend the service period	Ordinal categorical variable
Core independent variable	Training support	sup_train	Volunteer satisfaction with pre-service and in-service training: 1 = average; 2 = basically satisfied; 3 = very satisfied	Ordinal categorical variable
	Public service motivation	mot_public	Public Service Motivation (mot_public): summative index ranging from 0 to 3, based on the sum of three motivational items (“responding to national calls,” “loving hometown,” “passion for volunteer work”). Higher scores indicate stronger public service motivation.	Ordinal categorical variable
Mediating variable	Job wellbeing	Job wellbeing	Volunteers' overall evaluation of their volunteer work: 1 = not very satisfied; 2 = average; 3 = basically satisfied; 4 = very satisfied	Ordinal categorical variable
Control variables	Policy awareness	sup_policy	Assigned value based on volunteers' awareness of 4 core policies: 1 point for knowing 1 policy, up to 4 points for knowing all 4 policies	Ordinal categorical variable
	Financial support	sup_econ	Whether subsidies meet basic living needs: 1 = yes; 0 = no	Dummy variable (0/1)
	Security support	sup_security	Whether the local project office has paid social insurance for you: 1 = yes; 0 = no	Dummy variable (0/1)
	Political status	Politics	1 = masses (non-party member); 2 = member of the Communist Youth League; 3 = party member (including probationary members)	Ordinal categorical variable
	Family economic status	family_econ	1 = strained; 2 = average; 3 = good; 4 = excellent	Ordinal categorical variable
	Family support	family_sup	Degree of family support for volunteer service: 1 = not very supportive; 2 = neutral; 3 = somewhat supportive; 4 = very supportive	Ordinal categorical variable
	Service type	Service	1 = core rural service program; 0 = general rural public service program	Dummy variable (0/1)

### 4.3 Empirical methods

The dependent variable in this study, “career retention intentions,” is an ordinal categorical variable (ranging from 1 to 4). The core explanatory variables and mediating variables are either ordinal categorical or dummy variables. Based on the requirements of the research hypotheses, ordinal logistic regression and the bootstrap method for mediation effect testing are employed. The specific formulas and logic are as follows:

#### 4.3.1 Main effect test

An ordinal logistic regression model (ologit) is used to examine the main effects of core variables on the career retention intentions.

First, the basic form of the model is specified. For the ordinal dependent variable *Y*_*i*_ (career retention intentions, taking values *j* = 1, 2, 3, 4), the cumulative probability *P* (*Y*_*i*_ ≤ *j*) is defined as the probability that the career retention intentions for the *i*th sample does not exceed level *j*. The core formula of the model is as follows:


$$\begin{array}{l} \log it\left[P\left(Y_{i}\leq j\right)\right]=\ln\;\left(\frac{P\left(Y_{i}\leq j\right)}{1-P\left(Y_{i}\leq j\right)}\right)=\alpha_{j}-\beta X_{i} \end{array}$$

Here, *j* = 1, 2, 3, 4 (since the four-level dependent variable corresponds to three cumulative probability equations, satisfying α_1_ <α_2_ <α_3_, where α_*j*_ represents the intercept term for the *j*_*th*_ equation). *X*_*i*_ denotes the vector of independent variables, encompassing the core explanatory variables (training support and public service motivation) and control variables (policy perception, economic support, family support, etc.). The vector β represents the regression coefficients of the independent variables, capturing the effect of the independent variables on the log of the cumulative odds ratio. Specifically, if β > 0, it indicates that a larger value of the independent variable is associated with a higher probability that the dependent variable falls into a higher category.

Second, a stepwise regression approach is adopted to construct a series of models, aiming to clearly isolate potential interference effects among variables.

Model 1 includes only control variables to examine their baseline impact on the career retention intentions.


$$\begin{array}{l} \log it\left[P\left(Y_{i}\leq j\right)\right]=\alpha_{j1}-\beta_{1}Control_{i} \end{array}$$

Model 2 incorporates the control variables and training support to examine the main effect of training support.


$$\begin{array}{l} \log it\left[P\left(Y_{i}\leq j\right)\right]=\alpha_{j2}-\beta_{2}Control_{i}-\gamma_{1}\sup\text{\_}train \end{array}$$

Model 3 incorporates the control variables and public service motivation to examine the main effect of public service motivation.


$$\begin{array}{l} \log it\left[P\left(Y_{i}\leq j\right)\right]=\alpha_{j3}-\beta_{3}Control_{i}-\gamma_{2}mot\text{\_}public \end{array}$$

Model 4 incorporates the control variables, training support, and public service motivation to examine the joint main effects of the core explanatory variables.


$$\begin{array}{l} \log it\left[P\left(Y_{i}\leq j\right)\right]=\alpha_{j4}-\beta_{4}Control_{i}-\gamma_{1}\sup\text{\_}train-\\ {\;\gamma }_{2}mot\text{\_}public \end{array}$$

Model 5 incorporates all variables (control variables, core explanatory variables, and mediating variables) and serves as the foundation for the mediation effect test.


$$\begin{array}{l} \log it[P(Y_{i}\leq j)]=\alpha_{j5}-\beta_{5}Control_{i}-\gamma_{1}^{'}\sup\_train-\\ \;\gamma_{2}^{'}mot\_public-\delta satisfact \end{array}$$

*Control*_*i*_ denotes the control variables, while $\gamma_{1}^{'}$ and $\gamma_{2}^{'}$ represent the regression coefficients of the core explanatory variables after including the mediating variable, and δ represents the regression coefficient of the mediating variable.

#### 4.3.2 Mediation effect test

The bootstrap method (with 1,000 resamples) was employed to examine the mediating role of job wellbeing across different pathways, thereby addressing the limitations of traditional stepwise regression approaches.

## 5 Empirical analysis and discussion of results

### 5.1 Main effect test results

This study employs a stepwise regression approach, sequentially incorporating control variables, core explanatory variables, and mediating variables into the models to gradually isolate potential confounding effects and clearly delineate the main effects of the core variables. The regression results are presented in Table 2. The pseudo *R*^2^ values for Models (1) through (5) exhibit a progressive increase, indicating a continuous improvement in model fit, with the direction of the core variables' effects being strongly consistent with theoretical predictions.

**Table 2:** Ordinal logistic regression results.

Variables	(1)	(2)	(3)	(4)	(5)
	Retention	Retention	Retention	Retention	Retention
sup_policy	0.060	0.010	0.070	0.023	−0.005
	(0.090)	(0.092)	(0.093)	(0.096)	(0.098)
sup_econ	0.491	0.410	0.439	0.373	0.271
	(0.300)	(0.296)	(0.297)	(0.292)	(0.283)
sup_security	−0.294	−0.139	−0.355	−0.211	−0.459
	(0.696)	(0.689)	(0.664)	(0.668)	(0.682)
Politics	0.059	0.085	0.086	0.115	0.106
	(0.205)	(0.212)	(0.204)	(0.211)	(0.212)
family_econ	−0.393^**^	−0.382^**^	−0.325^*^	−0.321^*^	−0.327^*^
	(0.189)	(0.191)	(0.193)	(0.194)	(0.196)
family_sup	0.879^***^	0.732^***^	0.819^***^	0.690^***^	0.504^***^
	(0.135)	(0.144)	(0.138)	(0.147)	(0.151)
Service	−0.083	−0.096	−0.113	−0.122	−0.194
	(0.228)	(0.231)	(0.228)	(0.231)	(0.229)
sup_train		0.550^***^		0.503^***^	0.361^**^
		(0.182)		(0.183)	(0.183)
mot_public			0.337^***^	0.311^***^	0.310^***^
			(0.103)	(0.104)	(0.104)
Job wellbeing					0.590^***^
					(0.174)
cut1	−1.154	−0.357	−0.795	−0.084	0.222
	(1.029)	(1.085)	(1.034)	(1.096)	(1.119)
cut2	1.014	1.852^*^	1.378	2.129^*^	2.480^**^
	(1.025)	(1.079)	(1.027)	(1.089)	(1.114)
cut3	3.108^***^	3.987^***^	3.519^***^	4.305^***^	4.715^***^
	(1.020)	(1.083)	(1.029)	(1.100)	(1.133)
*N*	369	369	369	369	369

Standard errors in parentheses.

^*^P < 0.1, ^**^P < 0.05, ^***^P < 0.01.

#### 5.1.1 Control variable test results

Model (1) reveals that family support (family_sup) is significantly positive at the 1% level (β = 0.879, *P* < 0.01), indicating that family encouragement and recognition serve as crucial external support for volunteers' career retention intentions. Conversely, family economic status (family_econ) is significantly negative at the 1% level (β = −0.393, *P* < 0.01), potentially because volunteers from wealthier families have more career options, resulting in higher opportunity costs for participating in rural revitalization voluntary services and thus exhibiting lower willingness to retention. The remaining control variables (e.g., policy perception, economic support) do not demonstrate statistical significance, suggesting their relatively weak influence on the career retention intentions.

#### 5.1.2 Main effect test results of core explanatory variables

First, the main effect of training support. In Models (2) and (4), the coefficients for training support (sup_train) are significantly positive at the 1% level (β = 0.550, *P* < 0.01; β = 0.503, *P* < 0.01), indicating that for each unit increase in training support, the odds of volunteers' career retention intentions increase substantially. Thus, Hypothesis H1 is supported. This finding aligns with the expectations of social exchange theory and the theory of planned behavior, suggesting that high-quality training not only provides volunteers with capacity-building resources but also enhances their perceived control over service tasks.

Second, the main effect of public service motivation. In Models (3) and (4), the coefficients for public service motivation (mot_public) are significantly positive at the 1% level (β = 0.337, *P* < 0.01; β = 0.311, *P* < 0.01), indicating that volunteers motivated by “responding to national calls, love for their hometown, and passion for voluntary work” demonstrate significantly higher career retention intentions compared to those without such motivation. Thus, Hypothesis H2 is supported. This result corroborates the core proposition of public service motivation theory, suggesting that intrinsic value identification serves as a fundamental driver of volunteers' career retention.

#### 5.1.3 Mediating variable test results

In Model (5), after incorporating job wellbeing, its coefficient is significantly positive at the 1% level (β = 0.590, *P* < 0.01), indicating that higher job wellbeing is associated with stronger career retention intentions among volunteers. Meanwhile, the coefficient for training support decreases from 0.503 in Model (4) to 0.361 in Model (5), with its significance level dropping from 1 to 5%. In contrast, the coefficient for public service motivation remains relatively stable (from 0.311 to 0.310). This pattern of changes preliminarily suggests that job wellbeing mediates the relationship between training support and career retention intentions, while no such mediating effect is evident in the pathway involving public service motivation. These findings provide initial evidence warranting further mediation analysis.

### 5.2 Mediation effect test results

To precisely identify the differential mediating role of job wellbeing, this study employed the bootstrap method (with 1,000 resamples and a fixed seed of 12,345) to conduct the mediation analysis. The results are presented in Table 3. The test focused on the significance of the indirect effect (a × b), with the core criterion being whether the 95% confidence interval includes zero: if the confidence interval does not contain zero, the mediation effect is considered significant; otherwise, it is not.

**Table 3:** Comparison of mediating effects across different pathways.

Effect type	(1) Public service motivation	(2) Training support
Indirect effect (a^*^b)	0.084	0.555^***^
	(0.080)	(0.206)
Direct effect (*c*)	0.328^***^	0.406^**^
	(0.103)	(0.191)
Total effect (a^*^b+c)	0.412^***^	0.961^***^
	(0.130)	(0.261)
Patha (X-M)	0.127	0.938^***^
	(0.112)	(0.213)
Pathb (M-Y)	0.665^***^	0.592^***^
	(0.173)	(0.179)
*N*	369	369

Standard errors in parentheses.

^*^P < 0.1, ^**^P < 0.05, ^***^P < 0.01.

#### 5.2.1 Mediating effect of the training support pathway

As clearly shown in Column (2) of Table 2, the coefficient for path a (sup_train → job wellbeing) is 0.938 (*P* < 0.01), and the coefficient for path b (job wellbeing → retention) is 0.592 (*P* < 0.01), both highly significant. The indirect effect (a × b) is 0.555 (*P* < 0.01), with its 95% confidence interval excluding zero, indicating a significant mediating effect. The direct effect (*c*') is 0.406 (*P* < 0.01), also significant. These results demonstrate that job wellbeing partially mediates the relationship between training support and career retention intentions, thereby supporting Hypothesis H3. The mediating effect accounts for 57.8% of the total effect, suggesting that more than half of the impact of training support on career retention intentions is transmitted through job wellbeing.

#### 5.2.2 Mediating effect of the public service motivation pathway

As shown in Column (1) of Table 2, the coefficient for path a (mot_publ → job wellbeing) is 0.127 (*P* > 0.1), which is not statistically significant. The indirect effect (a × b) is 0.084 (*P* > 0.1), with its 95% confidence interval containing zero, indicating the absence of a mediating effect. The direct effect (*c*') is 0.328 (*P* < 0.01), highly significant. These findings validate Hypothesis H4, namely that job wellbeing does not significantly mediate the relationship between public service motivation and career retention intentions, and that the influence of public service motivation on career retention intentions operates primarily through a direct effect. This profoundly reveals that volunteers with strong public service motivation are driven to remain by intrinsic value identification and conviction, rather than by satisfaction with working conditions—a finding highly consistent with the core tenets of public service motivation theory.

### 5.3 Discussion of results

Through testing both main effects and mediating effects, this study clearly reveals a “dual-pathway” differential mechanism underlying rural revitalization volunteers' career retention intentions. Drawing upon theoretical foundations and empirical findings, he discussion is elaborated as follows:

First, the “resource-psychology-behavior” transmission logic of exogenous training support. The partial mediation effect of training support corroborates the core tenets of social exchange theory. As an exogenous institutional resource, training support cannot directly translate into stable career retention intentions; rather, it must be transmitted through the psychological mechanism of job wellbeing, while simultaneously shaping volunteers' emotional resilience for grassroots work. High-quality training helps volunteers rapidly adapt to rural service contexts, enhance their service competencies, and reduce feelings of frustration during service delivery, consequently fostering positive evaluations of voluntary service (i.e., job wellbeing). This positive psychological experience, in turn, strengthens volunteers' perceptions of the “service input—psychological reward” balance, further consolidating their emotional resilience and ultimately promoting increased career retention intentions. Meanwhile, the significant direct effect of training support suggests that it can also directly enhance volunteers' career retention intentions by improving their perceived behavioral control and directly shaping their emotional resilience to cope with grassroots work challenges. These findings indicate that exogenous training support motivates volunteers through dual drivers—namely, direct capacity empowerment and indirect psychological empowerment, and indirect psychological empowerment via job wellbeing—providing empirical evidence for the effectiveness of formal training in enhancing young people's emotional resilience, job wellbeing and employability in the process of career transition from campus to grassroots work.

Second, the direct pathway from value identification to career retention, driven by endogenous psychological motivation. The direct effect of public service motivation underscores the explanatory power of public service motivation theory. Public service motivation—characterized by “responding to national calls, love for one's hometown, and passion for voluntary work”—represents a collective manifestation of volunteers' value identification with the rural revitalization strategy and their sense of national commitment. This intrinsic motivation possesses a transcendent quality, remaining unaffected by external psychological experiences such as job wellbeing. Even under challenging working conditions or less favorable service experiences, volunteers with high public service motivation maintain strong career retention intentions, driven by their beliefs in and sense of responsibility toward rural development. This finding profoundly illuminates the core driving force behind young volunteers' career retention in rural revitalization voluntary services: namely, compared to external institutional incentives, intrinsic value identification constitutes a more stable and enduring motivational factor.

Third, the differential mediating role of job wellbeing further clarifies the heterogeneous mechanism of training support and emotional resilience on career retention intentions. The results show that job wellbeing plays a key partial mediating role in the training support-career retention pathway, with a mediation contribution rate of 57.8%, which means that more than half of the effect of training support on career retention is transmitted through job wellbeing. The practical significance of these effects is noteworthy. For training support, a one-unit increase (e.g., moving from “average” to “basically satisfied”) increases the odds of being in a higher retention category by approximately 65% (OR = 1.65, derived from β = 0.503 in Model 4; Table 2). This fully confirms that training support for grassroots volunteers must focus on improving subjective job wellbeing to effectively translate capacity building into sustainable career retention intentions. In contrast, job wellbeing has no significant mediating effect in the public service motivation-career retention pathway, which is because the intrinsic emotional resilience formed by value identification is not dependent on subjective work satisfaction, and this finding enriches the research on the boundary conditions of emotional resilience on employ-ability in the grassroots work context.

#### 5.3.1 Practical significance of effect sizes

Beyond statistical significance, the practical magnitude of these effects merits attention. As shown in Model 4 of Table 2, the odds ratio for training support is 1.65 (OR = e∧(0.503)), meaning that a one-unit increase in training support satisfaction (e.g., moving from “average” to “basically satisfied”) is associated with a 65% increase in the odds of reporting a higher level of career retention intention. For public service motivation, the odds ratio is 1.36 (OR = e∧(0.311)), indicating a 36% increase. Furthermore, the mediation contribution of 57.8% for training support indicates that more than half of its total effect on retention operates through job wellbeing-a substantively large proportion with clear policy implications: improving training quality is not merely about skill transfer but fundamentally about enhancing volunteers' day-to-day work experience.

#### 5.3.2 International comparison and broader implications

The dual-path mechanism identified in this study—where exogenous training support operates through job wellbeing, while endogenous public service motivation operates directly—resonates with findings from international rural volunteer contexts. In Kenya, for instance, the high attrition rate among community health volunteers has been attributed to inadequate training, poor incentives, and a lack of recognition, while successful retention strategies emphasize both capacity building (exogenous) and a sense of purpose (endogenous) (Riang'a and Nyanja, 2024; Lusambili and Nyanja, 2021). In the World Agroforestry Centre's volunteer farmer trainer program, an 80% retention rate was achieved through a dual strategy that included financial mechanisms and entrepreneurial empowerment-echoing our finding that training support works both directly and indirectly (via wellbeing) to enhance retention. Across OECD countries, volunteer management frameworks highlight the importance of training, recognition, and alignment of volunteer motivations with organizational goals-principles that align closely with the “dual-path” logic identified here. While cultural, political, and institutional contexts vary significantly across regions-China's Western Program being a large-scale, state-led initiative, whereas many international programs are NGO-driven-the core finding that exogenous support and endogenous motivation operate through differentiated pathways appears to be transferable. This suggests that program designers worldwide should recognize that training support and intrinsic motivation are not interchangeable; they influence retention through distinct mechanisms and thus require distinct management strategies.

### 5.4 Robustness checks for public service motivation measurement

To address potential concerns regarding the measurement of public service motivation (PSM), we conducted a robustness check using an alternative binary specification. Specifically, a stricter binary variable (“at least two motives,” denoted psm) was created, coded as one if the volunteer selected at least two of the three motivational items (“responding to national calls,” “cherishing one's hometown,” and “passion for volunteer work”), and zero otherwise. This stricter criterion captures volunteers with stronger, more genuine value-driven public service motivation.

We then re-estimated the main effect models (Models 1–4) using this alternative PSM measure. The results are presented in Table 4.

**Table 4:** Ordinal logistic regression results (robustness check for psm measurement).

Variables	(1)	(2)	(3)	(4)	(5)
	Retention	Retention	Retention	Retention	Retention
sup_policy	0.060	0.010	0.075	0.027	−0.001
	(0.090)	(0.092)	(0.091)	(0.094)	(0.097)
sup_econ	0.491	0.410	0.451	0.384	0.285
	(0.300)	(0.296)	(0.298)	(0.293)	(0.285)
sup_security	−0.294	−0.139	−0.373	−0.225	−0.467
	(0.696)	(0.689)	(0.674)	(0.672)	(0.694)
Politics	0.059	0.085	0.093	0.119	0.110
	(0.205)	(0.212)	(0.203)	(0.210)	(0.213)
family_econ	−0.393^**^	−0.382^**^	−0.348^*^	−0.343^*^	−0.349^*^
	(0.189)	(0.191)	(0.191)	(0.192)	(0.194)
family_sup	0.879^***^	0.732^***^	0.851^***^	0.716^***^	0.532^***^
	(0.135)	(0.144)	(0.138)	(0.147)	(0.154)
Service	−0.083	−0.096	−0.096	−0.107	−0.176
	(0.228)	(0.231)	(0.227)	(0.229)	(0.228)
sup_train		0.550^***^		0.511^***^	0.372^**^
		(0.182)		(0.182)	(0.182)
psm			0.621^***^	0.572^***^	0.552^***^
			(0.208)	(0.208)	(0.209)
Satisfaction					0.579^***^
					(0.177)
cut1	−1.154	−0.357	−0.909	−0.188	0.109
	(1.029)	(1.085)	(1.033)	(1.086)	(1.115)
cut2	1.014	1.852^*^	1.262	2.026^*^	2.365^**^
	(1.025)	(1.079)	(1.027)	(1.080)	(1.110)
cut3	3.108^***^	3.987^***^	3.395^***^	4.194^***^	4.590^***^
	(1.020)	(1.083)	(1.028)	(1.091)	(1.129)
*N*	369	369	369	369	369

Standard errors in parentheses.

^*^P < 0.1, ^**^P < 0.05, ^***^P < 0.01.

As shown in Table 4, the coefficient for psm in Model 4 was positive and significant (β = 0.572, *P* < 0.01), consistent with the direction and significance of our main analysis using the summative index (0–3). This confirms that volunteers with stronger public service motivation have higher career retention intentions, and that this core finding is not sensitive to the specific measurement of PSM.

## 6 Conclusions, implications, and future directions

### 6.1 Research conclusions

Based on survey data from 369 university student volunteers participating in rural revitalization in Hubei Province-an in-depth regional case study of a typical central China province, this study integrates three classical theories to construct an analytical framework incorporating dual core variables. It empirically examines the mechanisms through which training support and public service motivation influence the career retention intentions. The following core conclusions are drawn: first, both training support and public service motivation exert significant positive effects on rural revitalization volunteers' career retention intentions, with the total effect of training support (0.961) substantially exceeding that of public service motivation (0.412), indicating that exogenous training support remains predominant in current volunteer incentive systems. Second, job wellbeing partially mediates the relationship between training support and career retention intentions, with a mediation contribution of 57.8%, suggesting that training support achieves indirect motivation through a “capacity empowerment → job wellbeing → career retention” pathway. Third, job wellbeing does not significantly mediate the relationship between public service motivation and career retention intentions; rather, public service motivation drives volunteer retention through a direct “value identification → career retention” pathway.

### 6.2 Practical implications

Based on these findings and aligned with the strategic demands of rural revitalization, the following differentiated volunteer motivation strategies are proposed to facilitate the development of stable and efficient volunteer teams.

First, optimize the training support system to strengthen both resource-based and psychological empowerment. Specifically, training content should be precisely tailored to rural revitalization needs, incorporating “general competencies plus specialized skills”—such as rural governance techniques, agricultural technology knowledge, and emergency response capabilities—to enhance relevance. Training delivery methods should be innovated through a “online micro-courses + offline practice + mentorship pairing” model to improve effectiveness. Additionally, feedback mechanisms for training outcomes should be established, enabling dynamic optimization of training content based on volunteers' experiences and work requirements, thereby enhancing job wellbeing through high-quality training.

Second, strengthen value-oriented guidance to activate the endogenous drive of public service motivation. During volunteer recruitment, priority should be given to selecting young individuals demonstrating value identification with “responding to national calls and loving one's hometown,” thereby solidifying the foundation of intrinsic motivation. Throughout the service process, volunteers' value identification should be reinforced through activities such as “rural revitalization exemplary presentations, hometown development achievement displays, and volunteer service story sharing.” Furthermore, a spiritual incentive system should be established, providing recognition through commendations and publicity for volunteers exhibiting high public service motivation and exceptional service outcomes, thereby amplifying the motivational effects of intrinsic drives.

Third, implement differentiated incentive strategies that accommodate both exogenous support and endogenous motivation. For volunteers with relatively weaker public service motivation, emphasis should be placed on optimizing exogenous institutional supports—including training and economic security—thereby enhancing their willingness to continue participating through improved work experiences. For volunteers with stronger public service motivation, priority should be given to strengthening value-oriented guidance and spiritual incentives, providing platforms for value realization, and fully unleashing the driving force of intrinsic motivation.

### 6.3 Limitations and future research directions

While this study contributes meaningful findings, several limitations warrant acknowledgment. First, the data source exhibits regional specificity, covering only Hubei Province. While Hubei is a typical central China region facing core rural revitalization challenges, and the Western Program is nationally standardized, we do not claim that our findings generalize to all volunteer programs in China. Instead, we position this study as an in-depth regional case study that provides internally valid evidence for the proposed dual-path mechanism. Future research could conduct cross-provincial comparative studies to analyze heterogeneity across different regions. Second, this study employs cross-sectional data, which cannot capture dynamic changes in variables. Future investigations could utilize panel data for longitudinal research, exploring the long-term evolutionary relationships among institutional support, public service motivation, and willingness to continue participating. Third, this study does not extensively examine differential effects across heterogeneous groups. Future research could conduct subgroup analyses based on dimensions such as gender, service specialization, and service duration to further refine the conclusions. Fourth, this study does not test interaction effects; future research could empirically examine the synergistic motivational effects of institutional support and public service motivation by constructing interaction terms. Fifth, this study used a single-item measure of job wellbeing. While single-item global measures of wellbeing have been validated in prior research (Wanous et al., 1997) and are appropriate for large-scale policy evaluation contexts, they lack the nuance of multi-dimensional scales. Future research could employ more comprehensive wellbeing instruments, such as the WHO-5 WellBeing Index or the Utrecht Work Engagement Scale (UWES), to further refine our understanding of the relationship between job wellbeing and career retention intentions.

Future research should address these limitations while also exploring new contexts such as digital rural construction and civilized practice in the new era, investigating the impact of emerging technologies and platforms on volunteers' willingness to continue participating. Such endeavors would provide more comprehensive theoretical support and practical pathways for the sustainable development of rural revitalization voluntary services.

## Data Availability

The original contributions presented in the study are included in the article/Supplementary material, further inquiries can be directed to the corresponding author.
