# Supplementary material for: Training support, public service motivation, and career retention intentions: the mediating role of job wellbeing among college graduate volunteers in China's rural revitalization
Source: Front Psychol. 2026 May 29;17:1835086. doi: 10.3389/fpsyg.2026.1835086 (PMC13260629; doi:10.3389/fpsyg.2026.1835086)
Supplement: Supplementary file 1 [file Table_1.docx]

Supplementary Material

# Supplementary Material

**Hubei Province University Student Volunteer Service Western Program (Rural Revitalization Program)Survey Questionnaire**

Dear Western Program (Rural Revitalization Program) Volunteer,

Greetings! To better understand and evaluate the implementation effectiveness of the Hubei Province University Student Volunteer Service Western Program (Rural Revitalization Program), and to continuously improve related work, we have designed this survey questionnaire. Your opinions and suggestions are very important to us and will help us better carry out university student volunteer service work. The information in this questionnaire will be kept strictly confidential and used solely for research purposes. Please feel free to respond. Thank you for your participation and support!

I. Basic Information

Does your family support your participation in the Western Program?

Strongly support

Somewhat support

Neutral

Somewhat oppose

Strongly oppose

Where is your family's residence located?

Urban area

Town

Rural area

What is your family's economic status?

Affluent

Good

Average

Financially strained

What are your main reasons for participating in the Hubei Province University Student Volunteer Service Rural Revitalization Program? (Multiple selections allowed)

Respond to the national call, serve the grassroots

Accumulate practical experience, enhance personal development

Strong affection for rural areas, desire to contribute

Seek new pathways for career development

Policy support and incentives (e.g., for graduate school entrance exams, civil service exams)

Temporarily alleviate current employment pressure

Passion for volunteer work

Cultivate abilities and skills, lay a foundation for future personal growth

Other, please specify: ___________

How did you learn about the Western Program? (Multiple selections allowed)

Western Program official website

"Western Volunteer Hub," "China Youth Volunteer" WeChat public accounts

University career services department and its website, WeChat public accounts, etc.

University Youth League Committee and its website, WeChat public accounts, etc.

Grade-level or class online groups

Academic advisors, faculty members

Senior students/alumni

Family and friends

Other, please specify: ___________

What is your political affiliation?

Member of the Communist Party of China (including probationary members)

Member of the Communist Youth League of China

Member of a democratic party

Non-affiliated

What is the classification of the university from which you graduated?

"Project 985" university

"Project 211" university (but not "Project 985")

"Double First Class" university (but neither "Project 985" nor "Project 211")

Regular undergraduate institution

Vocational/technical college

II. Volunteer Service Situation

Which service specialization(s) do you belong to? (Multiple selections allowed)

Rural Education

Service for Rural Construction

Healthy Villages

Grassroots Youth Work

Rural Social Governance

Other (please specify): ___________

Is your service post located in any of the following areas or regions?

National key rural revitalization assistance county

Village within a local key rural revitalization assistance county

Resettlement community for relocation

National rural governance model town

Other (please specify): ___________

What is your average daily working hours?

Less than 8 hours

8-10 hours

More than 10 hours

How many times per week do you typically work overtime?

0 times

1-2 times

3-4 times

5-7 times

What is your average duration per overtime session?

Never work overtime

Less than 1 hour

≥1 hour, <2 hours

≥2 hours, <3 hours

≥3 hours, <4 hours

4 hours or more

How satisfied are you with your current post?

Very satisfied

Somewhat satisfied

Neutral

Somewhat dissatisfied

Very dissatisfied

Does the work you currently do bring you a sense of happiness?

Yes

No

Does the work you currently do bring you a sense of achievement?

Yes

No

Does the work you currently do make you feel stressed?

Very stressed, almost unbearable

Relatively stressed, but manageable

Stressed, but not significantly

Not stressed at all

Do you frequently travel to villages or on business trips?

Frequently travel on business

Occasionally travel on business

Never travel on business

What are the main work-related problems you encounter during your service? (Multiple selections allowed)

Unfamiliarity with work processes

Harsh working environment

Local language barriers

Insufficient professional knowledge or skills

Complex interpersonal relationships

Mismatch between personal abilities and job requirements

Lack of adequate training and guidance

Difficulty in resource integration and coordination

Heavy workload, time constraints

Frequent duty shifts

Low stipend, delayed payment

Personal value not realized

Other (please specify): ___________

What are the main life-related problems you encounter during your voluntary service? (Multiple selections allowed)

Lack of recreational resources

Poor accommodation conditions

Dietary inadaptability

Inconvenient transportation

Cost of living not aligned with stipend

Job instability

Uncertain future prospects

Romantic relationships affected by work

Perceived inferior compensation and development prospects compared to other programs

Other (please specify): ___________

What is your approximate monthly living expense?

Less than 1000 RMB

1000-1500 RMB

1501-2000 RMB

2001-3000 RMB

More than 3000 RMB

What was your originally planned service period for participating in this volunteer program?

One year

Two years

Three years

Based on your experience during this period, what is your current intention to continue participating in volunteer service?

Very willing, hope to extend service period

Relatively satisfied, but the original planned service period is sufficient

Still considering, need more time to decide

Somewhat dissatisfied, hope to shorten or terminate service period early

Other (please specify): ___________

Are you aware that the Volunteer Service Certificate and Service Appraisal Form are important credentials for volunteers to enjoy relevant policies upon service completion?

Yes

No

What awards have you received during your service period?

International-level award

Provincial-level award

Municipal-level award

County-level or below award

No awards received

III. Training Situation

What types of support or training do you consider most important?

Professional skills training

Local language and culture training

Mental health counseling

Work methods and techniques training

Teamwork and communication skills training

Other (please specify): ___________

How many days of pre-service training did the local Project Management Office organize for you in total?

4 days or more

1-3 days

Not conducted

How satisfied are you with the pre-service training conducted by the Project Management Office?

Very satisfied

Somewhat satisfied

Neutral

Somewhat dissatisfied

Very dissatisfied

What suggestions or expectations do you have regarding the content of the pre-service training?

IV. Daily Management

After arriving at your service location, did you sign a tripartite service agreement with the local Project Management Office and the service unit?

Yes

No

Did you complete the second job registration as required (i.e., after arriving at the service county, confirming the service post in the Western Program National Information Management System)?

Yes

No

Are you aware of the relevant policies regarding the service period of Western Program volunteers and the recognition of this period as part of the length of service for seniority purposes?

Yes

No

Are you aware of the corresponding preferential policies for Western Program volunteers when applying for master's degree programs?

Yes

No

Are you aware of the preferential policies for Western Program volunteers regarding tuition compensation and student loan repayment?

Yes

No

Are you aware of the preferential policies for Western Program volunteers concerning professional title evaluation?

Yes

No

Can you receive notifications, messages, policies, and documents issued by the local Project Management Office in a timely manner?

Always

Most of the time

Occasionally

Not at all

Does the local Project Management Office under which you serve provide employment services for volunteers?

Yes

No

Not sure

Has the local Project Management Office under which you serve promoted local preferential employment policies and actively encouraged outstanding volunteers with completed service to stay and work locally?

Yes

No

Not sure

V. Financial Support

Are you able to receive your monthly work and living stipend as a Western Program volunteer on time?

Always

Usually

Occasionally not

Not at all

If your work and living stipend is delayed, which portion is typically delayed?

The portion disbursed by the Project Management Office is delayed

The portion disbursed by the service unit is delayed

Both portions are delayed

Not sure

Other

What is the usual reason for the delay in receiving your work and living stipend? (Multiple selections allowed)

Delay in operation by the county-level Project Management Office

Delay in operation by the provincial-level Project Management Office

Insufficient matching funds from local finance departments

Not sure

Other (please specify): ___________

Is there currently any portion of your work and living stipend that remains unpaid?

Yes, the starting month of unpaid stipend is: ___________

No

When is your work and living stipend usually disbursed?

Early in the current month

Mid-month

Late in the current month

Early in the following month

Mid-month of the following month

Late in the following month

Irregular schedule

Does your work and living stipend sufficiently cover your basic living needs at the service location?

Yes

No

Has the local Project Management Office under which you serve enrolled you in social insurance?

Yes

No, the Project Management Office did not enroll me

No, I voluntarily declined enrollment

Not sure

Does your service unit provide you with any additional work and living stipend?

Yes

No

Not sure

Has the local Project Management Office under which you serve purchased commercial insurance for volunteers, such as personal accident and critical illness insurance?

Yes

No

Not sure

Has the local Project Management Office under which you serve arranged health insurance for you?

Yes

No

Does the university from which you graduated provide you with any additional work and living stipend?

Yes

No

Not sure

VI. Future Plans

What are your plans after completing your service period? (Multiple selections allowed)

Stay in the township, continue deep engagement in rural revitalization

Return to urban areas for employment or further study

Prepare for civil service or public institution examinations

Start a business

Other (please specify): ___________

After completing your service period, would you support extending the service period as a means to alleviate employment pressure?

Strongly support

Support

Neutral

Oppose

Strongly oppose

After completing your service period, would you hope that local job opportunities are provided to allow you to settle in the countryside long-term?

Yes

Can consider

No

What are the main factors influencing your decision to stay and settle in the local area after completing your service period? (Multiple selections allowed)

Alignment with personal career ideals, aspiration to contribute to local economic and social development

Affection for the local culture, customs, and people

Desire to continue cultivating abilities at the grassroots level for better future development

Satisfactory local income and benefits that meet personal expectations

Hometown of origin

Already accustomed to local work and life rhythm, unwilling to change

Other (please specify): ___________

What kind of assistance do you hope Project Management Offices at various levels can provide to support your volunteer service work? (Multiple selections allowed)

Provide more training and skills enhancement opportunities

Increase financial support

Improve living conditions

Provide more communication platforms

Strengthen safety and security measures

Mental health and stress relief services

Career development planning and employment guidance

Enhance social influence and recognition

Other (please specify): ___________

Does the province where you intend to seek employment have specific civil service or public institution positions designated for Western Program volunteers?

Yes

No

Not sure

VII. Other (Work Suggestions)

What is your overall feeling about the volunteer service work?

Very satisfied, gained a lot

Relatively satisfied, but there is still room for improvement

Neutral, no strong feelings

Somewhat dissatisfied, somewhat disappointed

Very dissatisfied, hope to terminate early

What suggestions or comments do you have regarding the implementation of the Hubei Province University Student Volunteer Service Rural Revitalization Program?

Thank you for taking the time to complete this questionnaire. Your feedback is crucial for us to understand the current status, problems, and suggestions regarding the University Student Volunteer Service Western Program (Rural Revitalization Program), and will help us better support and serve the rural revitalization cause. We will carefully consider your opinions and suggestions and strive to create a better working and living environment for volunteers.

Supplementary Material should be uploaded separately on submission. Please include any supplementary data, figures and/or tables.

Supplementary material is not typeset so please ensure that all information is clearly presented, the appropriate caption is included in the file and not in the manuscript, and that the style conforms to the rest of the article.
